# Electron-driven and thermal chemistry during water-assisted purification of platinum nanomaterials generated by electron beam induced deposition

**DOI:** 10.3762/bjnano.9.10

**Published:** 2018-01-08

**Authors:** Ziyan Warneke, Markus Rohdenburg, Jonas Warneke, Janina Kopyra, Petra Swiderek

**Affiliations:** 1 University of Bremen, Faculty 2 (Chemistry/Biology), Institute of Applied and Physical Chemistry, Leobener Straße / NW 2, Postfach 330440, D-28334 Bremen, Germany; 2 Pacific Northwest National Laboratory, Physical Science Division, Richland, WA, USA; 3 Siedlce University, Faculty of Sciences, 4 Maja 54, 08-110 Siedlce, Poland

**Keywords:** carbon contamination, electron induced reactions, focused electron beam induced deposition, nanostructure purification, platinum precursor

## Abstract

Focused electron beam induced deposition (FEBID) is a versatile tool for the direct-write fabrication of nanostructures on surfaces. However, FEBID nanostructures are usually highly contaminated by carbon originating from the precursor used in the process. Recently, it was shown that platinum nanostructures produced by FEBID can be efficiently purified by electron irradiation in the presence of water. If such processes can be transferred to FEBID deposits produced from other carbon-containing precursors, a new general approach to the generation of pure metallic nanostructures could be implemented. Therefore this study aims to understand the chemical reactions that are fundamental to the water-assisted purification of platinum FEBID deposits generated from trimethyl(methylcyclopentadienyl)platinum(IV) (MeCpPtMe_3_). The experiments performed under ultrahigh vacuum conditions apply a combination of different desorption experiments coupled with mass spectrometry to analyse reaction products. Electron-stimulated desorption monitors species that leave the surface during electron exposure while post-irradiation thermal desorption spectrometry reveals products that evolve during subsequent thermal treatment. In addition, desorption of volatile products was also observed when a deposit produced by electron exposure was subsequently brought into contact with water. The results distinguish between contributions of thermal chemistry, direct chemistry between water and the deposit, and electron-induced reactions that all contribute to the purification process. We discuss reaction kinetics for the main volatile products CO and CH_4_ to obtain mechanistic information. The results provide novel insights into the chemistry that occurs during purification of FEBID nanostructures with implications also for the stability of the carbonaceous matrix of nanogranular FEBID materials under humid conditions.

## Introduction

Focused electron beam induced deposition (FEBID) produces solid nanomaterials with size down to the sub-10 nm regime by decomposing precursor molecules adsorbed on a surface under a tightly focused high-energy electron beam [1]. Applications of this technology range from repair of masks for photolithography [2] and the fabrication of AFM tips [1] to novel photonic [3–4] or plasmonically active [5] devices and sensor concepts [6]. Also, nanoscale structures grown by FEBID may possess promising magnetic properties [7–8]. However, metallic nanostructures produced by FEBID are often contaminated by considerable amounts of carbon, preventing them from fulfilling their desired functionality [1,9].

The main source of this impurity is the precursor itself that is used for the process. FEBID precursors typically contain atoms of the desired solid material and organic ligands that enhance their volatility. Metal organic precursors are thus used to fabricate metallic deposits. In the ideal case, a pure metal should remain at the surface while the organic ligands decompose into volatile products that are pumped away. However, this is usually not the case and material from the ligands tends to be incorporated in the deposit and thus deteriorates its physical properties [1,9–11].

**Figure 1:**
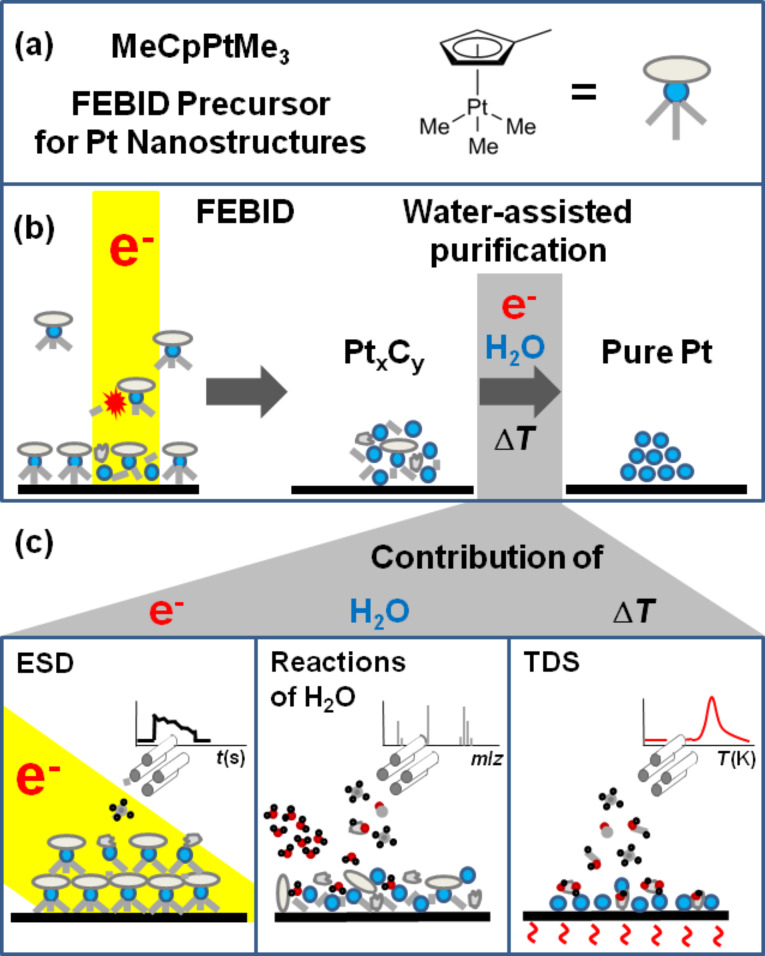
Molecular structure of the FEBID precursor trimethyl(methyl-cyclopentadienyl)platinum(IV) (MeCpPtMe_3)_ (a), schematic of the FEBID process and the subsequent water-assisted purification [12] of a deposit produced from MeCpPtMe_3_ (b), and mass spectrometric desorption experiments applied in this work to obtain insight in the underlying chemical reactions of the water-assisted purification process (c).

Trimethyl(methylcyclopentadienyl)platinum(IV) (MeCpPtMe_3_, Figure 1a) is widely applied as precursor for deposition of Pt because of its very favourable vapour pressure and good stability [1,10]. However, MeCpPtMe_3_ is a notoriously bad precursor for FEBID because it typically yields deposits with Pt content below 20% [1,9–11], which is insufficient for applications calling for pure metallic properties. In fact, deposits produced by FEBID from MeCpPtMe_3_ have a nanogranular structure, i.e., they consist of nanoscale Pt particles embedded in a carbonaceous matrix [12]. While FEBID-based PtC metal-matrix nanocomposites can serve, for instance, as transducing elements for humidity sensing [13], other applications call for high electrical conductivity. Therefore, different purification processes have been devised to turn the material into high-purity Pt [9,11,14–16]. In particular, post-deposition treatment with O_2_ at elevated temperature [9], the simultaneous exposure of the deposit to O_2_ and further electron irradiation [14–16], as well as FEBID of MeCpPtMe_3_ performed in the presence of O_2_ [15] have succeeded in yielding deposits with significantly improved Pt content [9] or even pure but often severely porous deposits [14–16].

It was recently shown that carbon layers in the presence of H_2_O vapour and graphene onto which a thin ice layer has been condensed can be efficiently etched by an electron beam [3,17]. Using a similar approach (Figure 1b), carbon-rich deposits produced from the ‘notoriously bad’ precursor MeCpPtMe_3_ can be efficiently purified by a post-deposition electron-beam treatment in the presence of H_2_O vapour whereby a densely packed carbon- and oxygen-free Pt material is obtained [12]. H_2_O is a favourable purification reagent also because it is typically less aggressive towards electron gun filaments than O_2_. If this purification procedure can be fully understood, controlled, and transferred to FEBID deposits produced from other carbon-containing precursors, the portfolio of compounds suitable for FEBID would widen enormously. This would provide a new perspective to overcome the challenges that FEBID faces regarding the generation of pure metallic nanostructures. However, to reach this goal, a detailed understanding on the molecular level is required. The previous study [12] reported the purification process on a phenomenological level and did not provide insight in its underlying chemical reactions. It could only assume that these reactions involve the conversion of the initial carbon material to small volatile products (CO, CO_2_, CH_x_) that, in the case of the FEBID deposit purification process, diffuse to the surface and desorb [12].

Here, we provide first insights on a molecular level into the underlying chemistry of this water-assisted purification process. We combine experiments on electron-stimulated desorption (ESD) and isothermal desorption of volatile products upon dosing of H_2_O with subsequent thermal desorption spectrometry (TDS). These experiments (Figure 1c) allow us to identify the volatile products, to distinguish between electron-driven reactions and contributions of thermal chemistry and to obtain insight into some mechanistic aspects of the water-assisted purification process. Our results provide a fundament for the molecular understanding and rational control of FEBID nanostructure purification using water as process gas [12] with additional implications for the stability of the carbonaceous matrix of nanogranular FEBID materials under humid conditions as relevant, for instance, in applications such as humidity sensing [13].

## Results and Discussion

### Thermal reactions between intact MeCpPtMe_3_ and H_2_O

**Figure 2:**
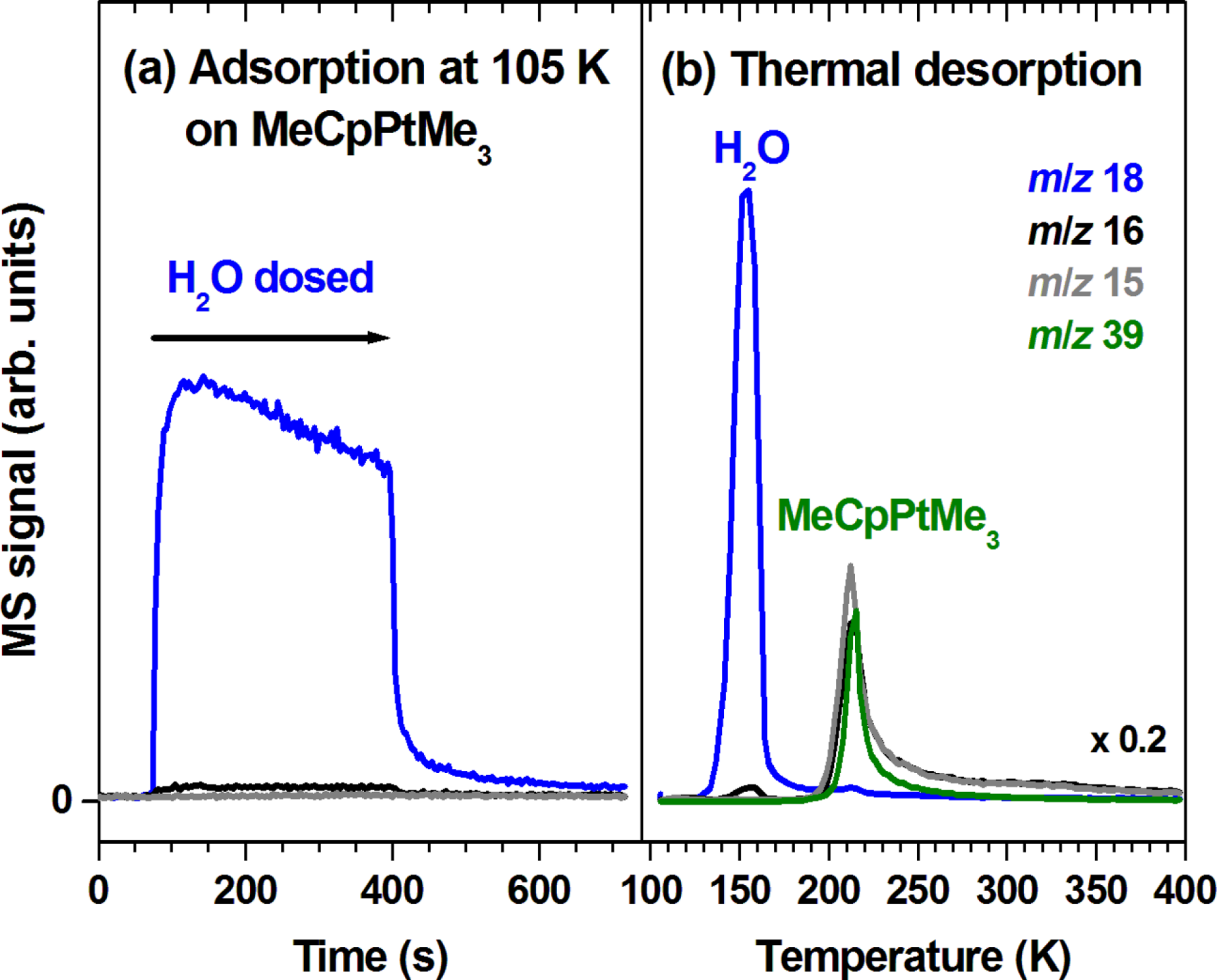
(a) MS signals recorded during leaking of H_2_O onto a film of MeCpPtMe_3_ with thickness corresponding to 30 monolayers deposited on a Ta substrate held at 105 K. The amount of H_2_O vapour applied in this experiment was the same as that used for depositing the MeCpPtMe_3_ multilayer film. The *m*/*z* ratios 18, 16, and 15 were recorded to monitor leaking of H_2_O as well as possible formation of CH_4_. For CH_4_, the relative intensity of the *m*/*z* ratios 16 and 15 amounts to 1:0.9 [10]. (b) TDS experiment performed subsequently on the same sample. Here, *m*/*z* 39 (C_3_H_3_^+^) is also included as a characteristic MS signal of MeCpPtMe_3_ (see [11]).

In ALD processes for deposition of Pt performed at 100 °C and consisting of alternating cycles of precursor and O_2_ dosing, MeCpPtMe_3_ reacts with surface hydroxyl groups produced during a preceding O_2_ exposure half-cycle whereby Pt–O bonds are formed and CH_4_ is released [18–19]. The analogous reaction between MeCpPtMe_3_ and H_2_O does not contribute noticeably in our low temperature (105 K) experiments as verified by exposing a condensed layer of the precursor to H_2_O vapour. During leaking of H_2_O (Figure 2a), generation of CH_4_ was indeed not seen. In a subsequent TDS experiment (Figure 2b), the desorption of H_2_O and intact MeCpPtMe_3_ (compare Supporting Information File 1, Figure S1) was observed at 150 K and 210 K, respectively. We note that at temperatures between 250 K and 350 K slightly increased intensities of the methane signals were detected. These amounts are, however, negligible in comparison to reaction products formed under electron irradiation, as shown in the following.

### Electron-induced degradation of multilayer condensed films of MeCpPtMe_3_

**Figure 3:**
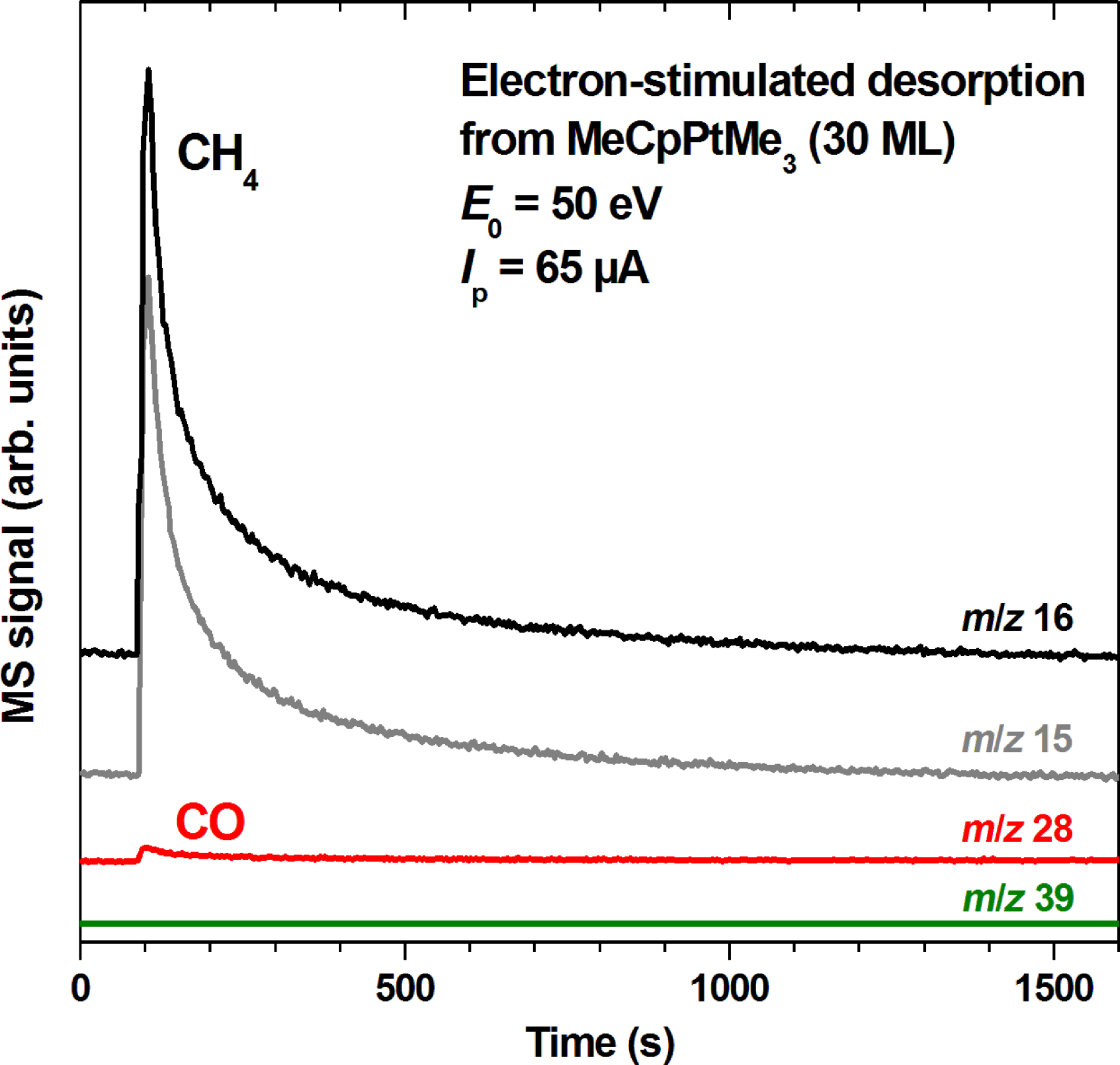
Electron-stimulated desorption from a 30 layer film of MeCpPtMe_3_ on a Ta substrate held at 105 K. An incident electron current *I*_p_ of 13 μA/cm^2^ was applied at *E*_0_ = 50 eV. The *m*/*z* ratios 16 and 15 were recorded to monitor formation of CH_4_ for which the relative intensity of the *m*/*z* ratios 16 and 15 amounts to 1:0.9 [10]. The *m*/*z* ratio 28 indicates desorption of CO and of traces of ethane (see Supporting Information File 1, Figure S2), while *m*/*z* 39 (C_3_H_3_^+^) is also included as a characteristic MS signal of MeCpPtMe_3_ (see [11]).

Electron-stimulated desorption was first measured from multilayer condensed films of MeCpPtMe_3_ without added H_2_O as reference for subsequent experiments (Figure 3). In accord with previous results [10], material is removed from the layer exclusively in form of CH_4_ molecules. This is obvious from the intensity ratio of 1:0.9 of the MS signals recorded at *m*/*z* 16 and 15 which is characteristic of CH_4_ thus excluding noticeable contributions of CH_3_ radicals that would lead to additional intensity of the *m*/*z* 15 signal. It was proposed previously that the exclusive observation of CH_4_ can be explained by reaction of desorbing CH_3_ radicals with hydrogen from the walls of the UHV chamber that occur before CH_3_ can reach the mass spectrometer [11]. Based on previous results from our setup, we can exclude such an artifact. In fact, ESD of CH_3_ was clearly observed from condensed layers of acetone and acetylacetone [20] in the same setup used also for the experiments described here.

Beside the predominant CH_4_, a small desorption signal with *m*/*z* ratio 28 is seen at the start of irradiation (Figure 3). It was identified as a minor CO background signal with equally small contributions of ethane (C_2_H_6_) according to a mass spectrum recorded during the initial stages of electron exposure (Supporting Information File 1, Figure S2). The very small quantity of C_2_H_6_, confirms again the previous conclusion [10] that recombination of CH_3_ ligands dissociating from the precursor is not a relevant reaction in the electron-induced degradation of MeCpPtMe_3_ layers. In addition, the *m*/*z* 39 trace gives evidence that ESD of the precursor is negligible.

According to previous gas phase experiments [21], the dominant fragmentation proceeding via dissociative electron attachment (DEA) leads to loss of only one neutral CH_3_ ligand. DEA occurs at low electron energies characteristic of the secondary electrons that are released in large numbers under impact of a high energy primary electron beam and are therefore thought to make major contributions to deposit formation. Assuming that the ligand material remaining behind after loss of the first CH_3_ is embedded in the deposit, DEA thus explains the deposit composition of Pt/C = 1:8 that was obtained when the precursor was decomposed at 180 K by electron exposure in UHV, i.e., without contributions of residual vapours to deposit formation [10]. From this perspective [11], it is surprising that only CH_4_ desorbs during electron irradiation and not CH_3_ itself. The previous ESD data on MeCpPtMe_3_ [10] revealed a linear increase of the CH_4_ yield as function of coverage. This was ascribed to an intramolecular reaction as origin of CH_4_ which is supported by the very small amount of C_2_H_6_ production as also observed here. The latter would be expected to result from recombination of CH_3_ radicals that have dissociated from the precursor. When electron irradiation is performed at *E*_0_ well above the ionization threshold, as in the present and previous [10] surface studies, precursor fragmentation is driven by electron impact ionization (EI). In this regime, loss of CH_4_ from the precursors has in fact been observed in gas phase mass spectrometry [11,20]. Nonetheless, the abundances of specific fragment ions observed in the positive ion mass spectra suggest that fragmentation does not exclusively produce CH_4_ but also some CH_3_ [21]. ESD of CH_3_ from a condensed layer of MeCpPtMe_3_ during electron exposure would thus again be anticipated.

Considering these electron-induced dissociation reactions of MeCpPtMe_3_, trapping of fragments in the precursor layer may provide a more convincing explanation for the lack of CH_3_ in ESD. Such an effect was observed previously in the case of acetylacetone where gas-phase experiments demonstrated that electron-induced loss of CH_3_ is significant in the monomers but is completely suppressed in the dimer and ESD of CH_3_ from a condensed layer of the compound is also weak [21]. In the case of MeCpPtMe_3_, the CH_3_ radical may add efficiently to the unsaturated cyclopentadienyl ligand or abstract a hydrogen atom from a second ligand. In the second case, abstraction from a CH_3_ ligand would yield an intermediate with Pt=CH_2_ unit, i.e., a carbene ligand. Such intermediates are akin to species occurring in dehydrogenation reactions of CH_3_ groups on Pt surfaces [22] and have also been observed in the gas phase following reactive interaction between Pt^+^ ions and CH_4_ [23].

Unfortunately, absolute cross section data for DEA and EI of MeCpPtMe_3_ as well as a detailed understanding of the fragmentation mechanisms following EI do not exist to the best of our knowledge. Furthermore, neutral dissociation (ND), a non-resonant process with a threshold comparable to EI and leading to the formation of uncharged fragments, may also play a role in the observed CH_4_ production. However, data on ND are generally difficult to obtain and do not exist so far for MeCpPtMe_3_. A complete set of such cross section data supplemented by theoretical studies of the electron-induced dissociation and of subsequent reactions of the resulting fragments would help to better understand which of the initiating electron-precursor interactions is more likely to dominate deposit formation.

### Interplay of electron-induced and thermal degradation of MeCpPtMe_3_

**Figure 4:**
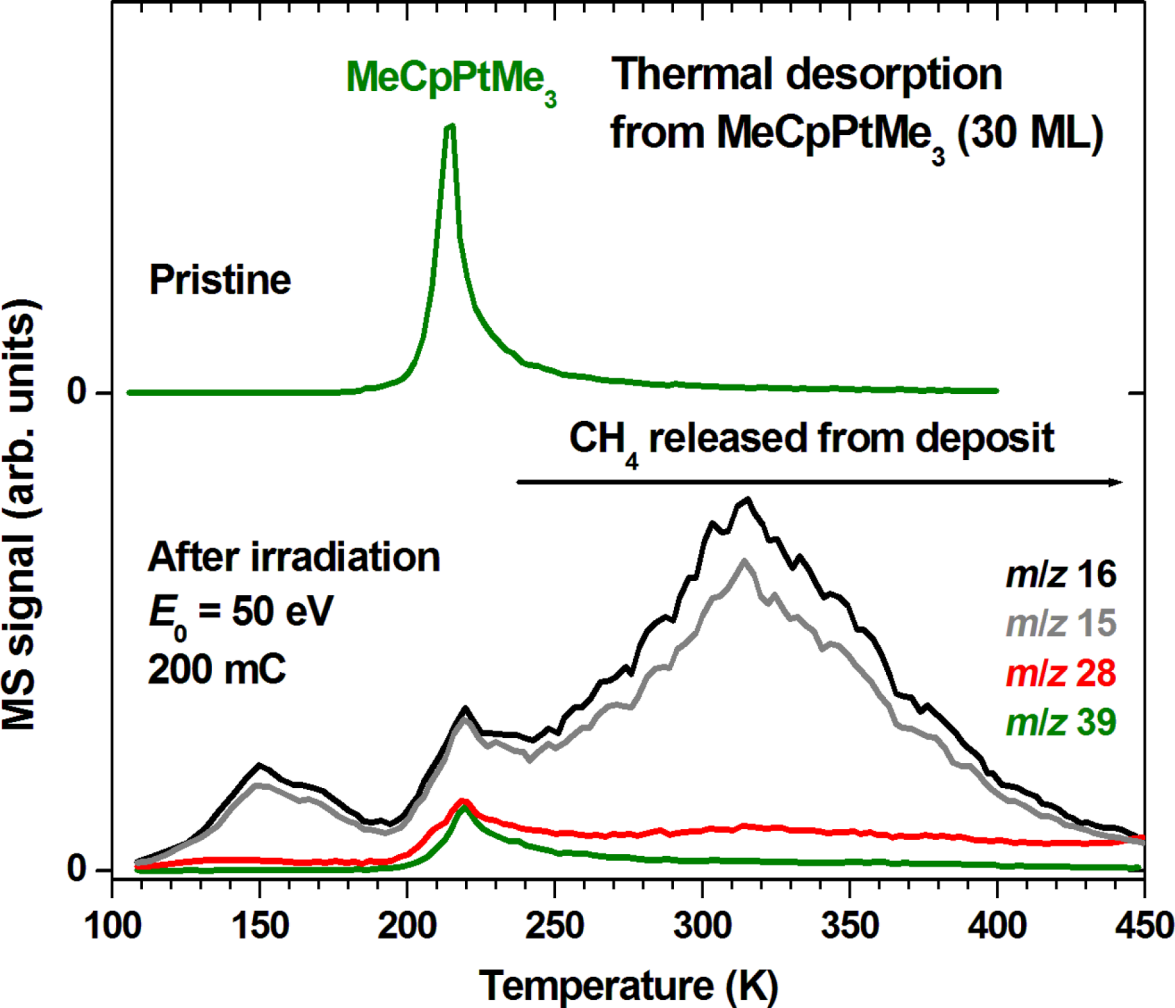
Thermal desorption spectra recorded from a 30 layer film of MeCpPtMe_3_ as deposited at 105 K on a Ta substrate (top) and from the MeCpPtMe_3_ film prepared for the experiment shown in Figure 3 but after a total electron exposure of 40 mC/cm^2^ at *E*_0_ = 50 eV (bottom). The *m*/*z* ratios 16, and 15 were recorded to monitor the possible formation of CH_4_ for which their relative intensities amount to 1:0.9 [10]. The *m*/*z* ratio 28 is included to monitor possible release of C_2_H_6_ and *m*/*z* 39 (C_3_H_3_^+^) is included as a characteristic MS signal of MeCpPtMe_3_ (see [11]). Only *m*/*z* 39 is shown for clarity in the case of the fresh layer (top) where other desorption signals were absent (see Supporting Information File 1, Figure S1).

As shown in Figure 2 and Supporting Information File 1, Figure S1 and in accord with previous results [10], non-irradiated MeCpPtMe_3_ desorbs in UHV without noticeable thermal decomposition. To investigate the contribution of thermal decomposition to the FEBID process, a TDS experiment was performed after extensive irradiation, i.e., at a time when the ESD signals of CH_4_ had decayed approximately to the baseline level (Figure 4). As shown previously [10], changes in oxidation state of Pt, loss of carbon, and the complete loss of the infrared CH stretching bands proceed on the same time scale as the decay of CH_4_ signals in ESD. Therefore, the complete decay of CH_4_ desorption appears to indicate that the precursor layer has been fully converted to a deposit as also obtained in a FEBID process. We note that such fully electron-degraded layers were also used to study the reactions underlying the water-assisted purification process (see following sections).

Figure 4 reveals that thermal reactions in fact occur in the degraded precursor layers. In the case of the irradiated sample (Figure 4, bottom), a small desorption peak near 220 K for *m*/*z* 39 and 28 suggests that a certain amount of precursor or a chemically very similar and equally volatile species was left on the substrate, despite the fact that ESD of CH_4_ had ceased. Attenuation of the electron beam in the relatively thick MeCpPtMe_3_ layer used in the present experiment may have led to incomplete conversion of deeper layers and can thus explain the remaining precursor signal. More important, however, is the thermal desorption of additional CH_4_ over a wide temperature range as evident from the TDS curves recorded at *m*/*z* 15 and 16. These desorption signals were absent in non-irradiated layers (Supporting Information File 1, Figure S1). The desorption signals around 150 K and at the desorption temperature of MeCpPtMe_3_ can be explained by release of some CH_4_ that was retained in the thick layer at 105 K. In contrast, the release of CH_4_ above 230 K points to thermal decomposition of less volatile products formed during electron irradiation of MeCpPtMe_3_. After desorption of the precursor, these products remain on the surface and undergo thermal reactions as the temperature is further increased. The signal *m*/*z* 28 observed in the same temperature range and reaching up to 450 K points to the formation of C_2_H_6_ or C_2_H_4_ as a side product of this thermal decay. This result gives evidence that decomposition of the precursor is not only an electron-driven process but also proceeds via additional thermal chemistry.

Such thermal chemistry that assists the removal of ligands from the precursor has been described before. In particular, previous surface science studies on the FEBID precursor Pt(PF_3_)_4_ have shown that the initial electron-induced fragmentation leads to loss of one intact PF_3_ ligand while further irradiation merely removes additional fluorine [24]. In contrast, more intact ligands are removed by increasing the temperature resulting in a deposit with higher Pt content [25]. Here we show that the contributions of electron-induced and thermal reactions to precursor decomposition can also be deduced from ESD and TDS experiments. From the MS intensities during ESD (Figure 3) and post-irradiation TDS (Figure 4) we can estimate that in the case of MeCpPtMe_3_ roughly 1.5 times more CH_4_ desorbs as a consequence of thermal processes than during the previous irradiation at 105 K. About half of this thermal CH_4_ release has occurred up to room temperature alone thus making a significant contribution to deposit formation. In addition, the further thermal decomposition extending up to 450 K is relevant to purification by post-deposition thermal purification processes. This result shows that a combination of ESD and TDS can provide an estimate of the relative contribution of electron-induced and thermal reactions in a given FEBID process.

### Water- and electron-induced reactions in electron-degraded multilayers of MeCpPtMe_3_

The fact that FEBID deposits produced from MeCpPtMe_3_ can be purified by electron irradiation in the presence of H_2_O vapour [12] implies that as a consequence of electron exposure the deposit reacts readily with H_2_O. To resolve under which conditions this reactivity sets in, thin condensed layers of MeCpPtMe_3_ were degraded by electron exposure to serve as model for a FEBID deposit. Specific amounts of H_2_O were then dosed onto the electron-degraded precursor layers held at 110 K. At this temperature, H_2_O condenses on the substrate. Reactions were monitored by measuring MS signals of neutral products that desorbed from the surface during dosing of H_2_O and during subsequent or simultaneous electron exposure. Also, the effect of annealing was investigated.

**Figure 5:**
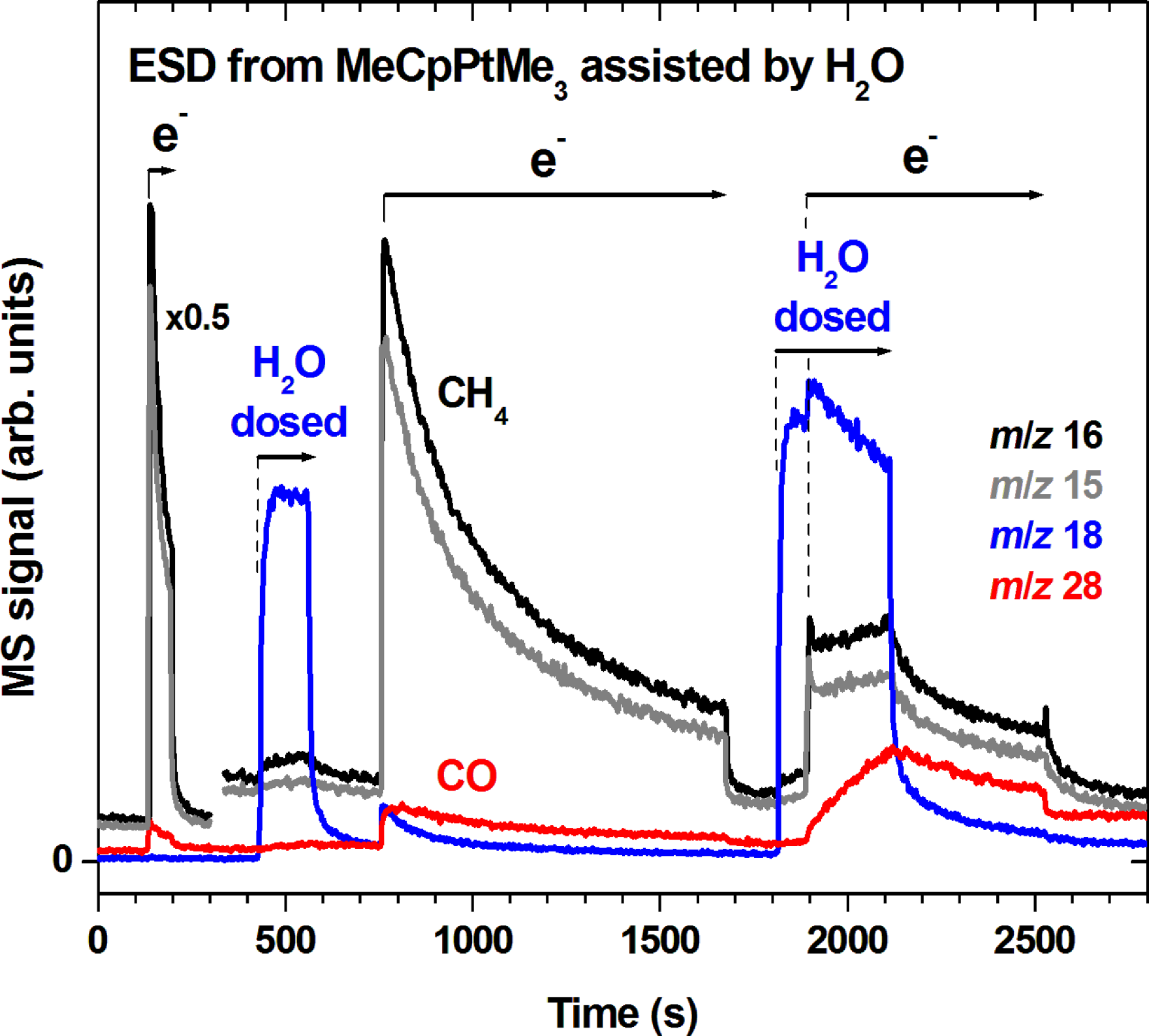
MS signals recorded during repeated electron exposures (e^−^) and leaking of H_2_O (H_2_O dosed) onto a 30 layer film of MeCpPtMe_3_ deposited and held at 105 K. During the first H_2_O leaking, the amount of vapour was 50% of that used for depositing the MeCpPtMe_3_ multilayer film. For the second leaking, the amount of H_2_O vapour was increased by a factor of two. During the three electron irradiation periods, exposures of 1.04 mC/cm^2^ (start at 130 s, *I*_p_ = 17 µA/cm^2^), 20 mC/cm^2^ (start at 750 s, *I*_p_ = 22 µA/cm^2^), and 14 mC/cm^2^ (start at 1900 s, *I*_p_ = 22 µA/cm^2^) were applied at *E*_0_ = 31 eV. A lower *E*_0_ as compared to Figure 3 was applied here to degrade the layers more slowly and consequently achieve better control over the number of injected electrons. The *m*/*z* ratios 18, 16, 15, and 28 were recorded to monitor leaking of H_2_O as well as possible formation of CH_4_ (relative intensity of *m*/*z* ratios 16 and 15 amounts to 1:0.9 [10]) and CO. Note that *m*/*z* 16 also contains a minor fragment signal of H_2_O.

In a first experiment (Figure 5), a 30 monolayer film of MeCpPtMe_3_ was partially degraded by electron irradiation until ESD of CH_4_ had decreased to about 50% of its initial value. At this stage of decomposition, a significant percentage of the precursor molecules must have lost at least one of their methyl groups. We note that the same products desorb as seen in Figure 3 despite the lower electron energy applied here. This is in accord with the result of a previous study on the electron-induced decomposition of MeCpPtMe_3_ by high-resolution electron energy loss spectroscopy (HREELS) [26] which has revealed that the nature of the deposit is similar for *E*_0_ = 50 eV and 500 eV. H_2_O was dosed onto this partially degraded precursor layer without prior annealing. This led to desorption of only small amounts of CH_4_ (Figure 5, between 400 and 600 s) as evident from a slight increase of the *m*/*z* 15 signal. This indicates that precursor fragments can be hydrolyzed to some extent but this reaction is much less efficient for removal of organic material than the initial electron irradiation. When electron exposure was resumed (at 750 s), the ESD rate of CH_4_ jumped back to its previous value at the end of the first irradiation period indicating that no major changes had incurred to the precursor layer during dosing of H_2_O. Also, as H_2_O does not hinder desorption of CH_4_, it must either have diffused into the precursor layer or grown on top in porous form. The presence of H_2_O in the layer is obvious from an ESD signal at *m*/*z* 18 with moderate intensity that set in immediately with the start of irradiation and decayed afterwards. However, irradiation now also led to ESD of CO (see also Supporting Information File 1, Figure S3) with slightly delayed onset and a much slower decay as compared to H_2_O and CH_4_. Comparative blind experiments on ESD from the substrate without precursor layer (not shown) revealed a small background desorption signal in the *m*/*z* 28 curve akin to that seen during the initial electron irradiation in Figure 5. However, the characteristically slower decay of this signal during the second electron exposure in Figure 5 implies that this CO desorption is not a background effect but results from a reaction in the precursor layer during electron exposure in the presence of H_2_O.

Electron irradiation was switched off again when ESD of CH_4_ had decreased to about 10% of its initial value (at 1700 s). Again, dosing of H_2_O alone (starting at 1800 s) led to only a small desorption signal of CH_4_. In contrast, switching on electron exposure (at 1900 s) during dosing of H_2_O initiated an immediate onset and subsequent further increase in the ESD rate of CH_4_ and a continuous increase of the CO ESD rate while condensation of H_2_O proceeded. Here, the amounts of CO clearly exceeded the background levels observed in the blind experiment. The increase of the CH_4_ ESD rate with continuing dosing of H_2_O during the third irradiation period implies that H_2_O is a limiting factor in this reaction. When the supply of H_2_O was stopped again (at 2100 s), all MS signals leveled off slowly and more strongly after electron exposure was also stopped (at 2500 s).

**Figure 6:**
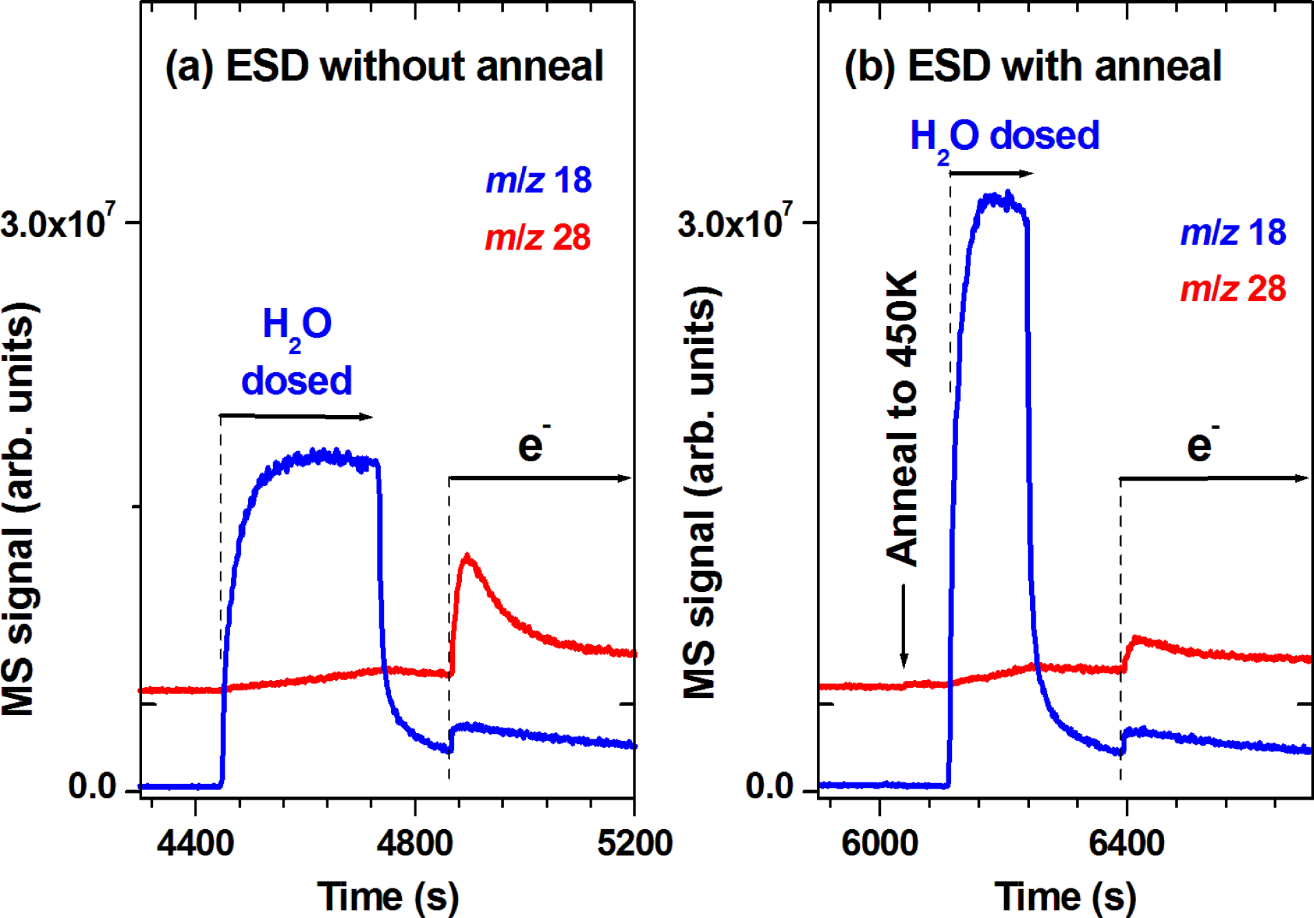
MS signals recorded during leaking of H_2_O (H_2_O) and electron exposure at *E*_0_ = 31 eV (e^−^) of a 30 layer film of MeCpPtMe_3_ deposited and held at 105 K. Prior to the processes steps shown here, the film was degraded by electron exposure at the same energy until ESD of CH_4_ had decayed. The amount of H_2_O vapour was the same as used for depositing the MeCpPtMe_3_ film. This procedure was performed (a) without and (b) with an annealing step to 450 K applied prior to leaking of H_2_O. The *m*/*z* ratios 18 and 28 are shown to visualize leaking of H_2_O and formation of CO. The dashed line marks the start of the irradiation period with (a) *I*_p_ = 10 µA/cm^2^ and (b) *I*_p_ = 12 µA/cm^2^.

ESD signals of CO during dosing of H_2_O but before the start of electron irradiation are too small to be visible in Figure 5. Therefore, the evolution of the CO signal has been investigated in a second set of experiments in which H_2_O was dosed only after ESD of CH_4_ had decayed nearly back to the background signal and irradiation was performed only after dosing of H_2_O (Figure 6). In fact, Figure 6 shows a small and continuous increase of the MS signal at *m*/*z* 28 during dosing of H_2_O. When electron irradiation was started after leaking of H_2_O without prior annealing, the desorption rate of CO increased strongly revealing again the action of H_2_O in contact with the deposit (Figure 6a). Careful inspection of the ESD signal reveals that the release of CO is somewhat delayed as compared to ESD of H_2_O implying that CO is formed by an intermediate that is then decomposed by electron exposure. We note that some minor production of CO_2_ was also observed and that a similar result was obtained in experiments performed in the same way but at 19 eV (not shown). As a difference, ESD of H_2_O was very small at 19 eV.

The experiments summarized in Figure 5 and Figure 6 show that the combined action of H_2_O and electron exposure is required for an efficient removal of organic material at cryogenic temperature. In fact, as seen in Figure 5, desorption of products in the presence of H_2_O alone proceeds at a relatively low rate even after a large fraction of MeCpPtMe_3_ has been decomposed by electron irradiation. This indicates that the intermediates that reside in the deposit after the initial electron-induced fragmentation of the precursor are not very reactive towards H_2_O at cryogenic temperature. The reason is difficult to trace without detailed knowledge of the structure of these species. However, it has been shown that H_2_O reacts only slowly with a model complex PtCH_2_^+^ prepared in gas phase [23] suggesting that such reactive intermediates may be relatively stable.

Electron-initiated reactions of H_2_O itself are a conceivable reason for the enhanced precursor or deposit degradation seen in Figure 5 and Figure 6 when the electron beam is switched on in the presence of H_2_O. Electron exposure of condensed H_2_O and clusters of H_2_O at *E*_0_ above the ionization threshold is known to induce the following proton transfer [27]


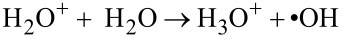


and thereby yields OH radicals. As MeCpPtMe_3_ tends to lose its cyclopentadienyl ligand when exposed to acidic conditions [28–29], a similar process is conceivable following ionization-induced proton transfer from H_2_O to the precursor


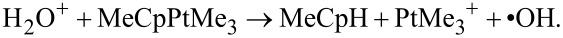


This would weaken the bonding between the negatively charged MeCp ligand and Pt thus exposing the latter to attack by H_2_O or the more reactive OH radical. This paves the avenues to oxidation of the ligands which is obvious here through formation of CO and even CO_2_. Such a scenario is supported by the fact that Pt complexes in aqueous solution are catalysts for oxidation of alkanes and other organic compounds [30].

Electron-induced reactions involving OH radicals have recently been discussed in relation to the electron-induced formation of ethanol in condensed mixtures of ethylene and H_2_O [31]. In this study, it was observed that product formation via reactions that are held to be maintained by a chain reaction involving OH radicals became only noticeable when the irradiated molecular layers were sufficiently thick. This effect was explained by deactivation of the OH radicals by capturing of thermalized electrons, an effect that can only occur near the surface and not below a depth roughly corresponding to the effective penetration depth of the electron beam. Purification of actual FEBID deposits was also observed to be more efficient at a greater depth within the layer [12] supporting that a contribution of OH radicals is a reasonable scenario in the context of deposit purification by electron exposure in the presence of H_2_O.

**Figure 7:**
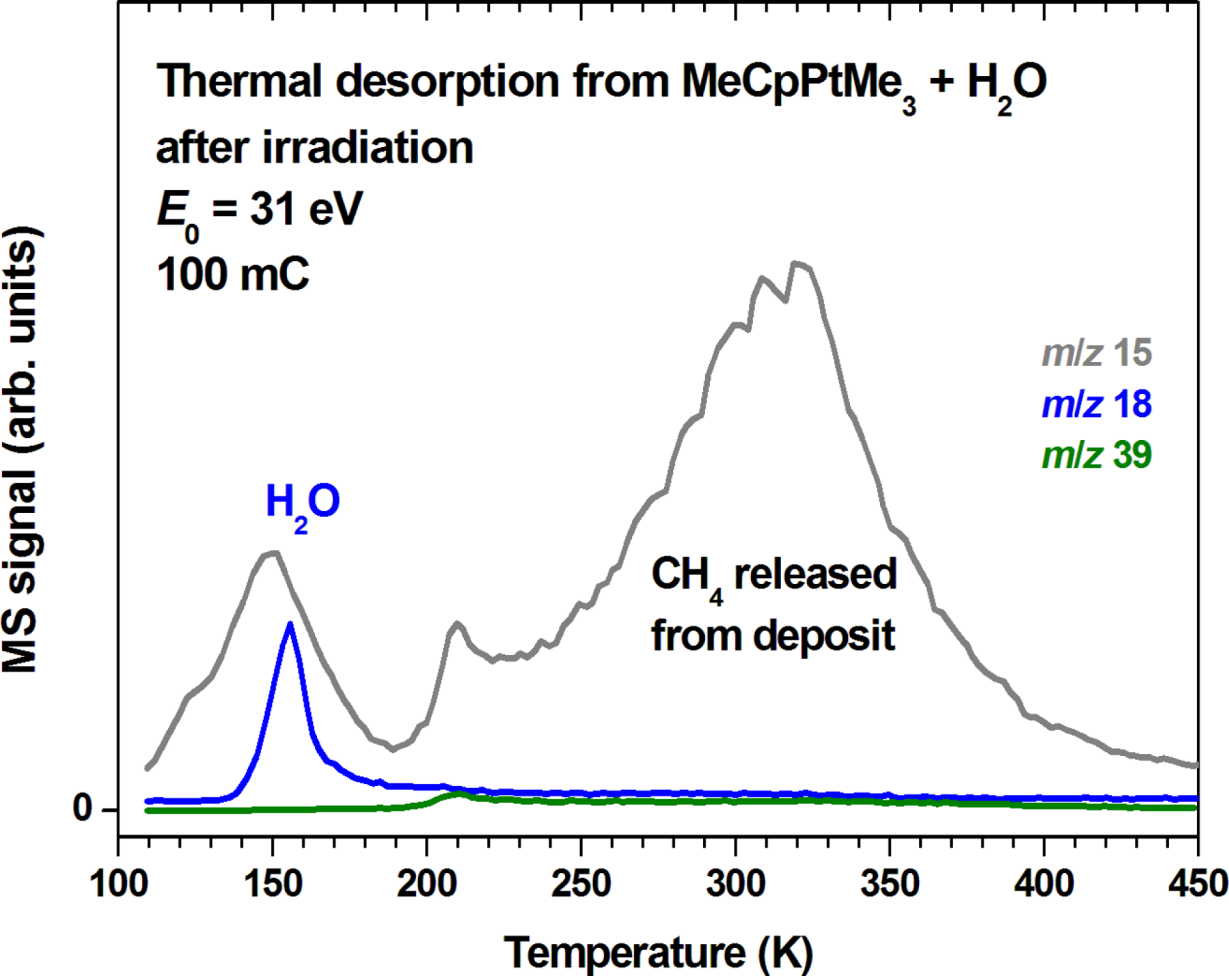
Thermal desorption spectra obtained after an electron exposure of 100 mC at *E*_0_ = 31 eV from a 15 layer film of MeCpPtMe_3_ condensed on a Ta substrate held at 105 K and covered by a threefold excess of H_2_O. The ratios *m*/*z* 18 and *m*/*z* 15 were recorded to monitor desorption of remaining H_2_O as well as possible formation of CH_4_. The *m*/*z* ratio 39 (C_3_H_3_^+^) is included as a characteristic signal of MeCpPtMe_3_ and of MeCpH that may possibly be released from the decomposed layer.

When the sample was annealed by heating to 450 K prior to dosing of H_2_O, significantly less CO desorbed during subsequent electron exposure (Figure 6b). This can be traced back to loss of material during the annealing as seen in TDS data (Figure 7). As in Figure 4, additional thermal decomposition is witnessed by desorption of further organic material (*m*/*z* 15) around the desorption temperatures of H_2_O (150 K) and remaining precursor (210 K) as well as, more strongly, above 220 K with maximum rate around room temperature. Desorption has again mostly ceased at 450 K in line with the earlier result showing that complete reduction of MeCpPtMe_3_ in CVD processes assisted by H_2_ can be achieved just below 400 K [19]. We conclude that too little material is left at the surface to gain deeper insight in the purification process when only a single multilayer film of MeCpPtMe_3_ has been decomposed.

We note that desorption of MeCpH was not observed although proton transfer to the precursor is antipicated when MeCpPtMe_3_ is irradiated in the presence of H_2_O. This is obvious from a lack of desorption signal below the precursor desorption temperature in the *m*/*z* 39 data shown in Figure 7. Unlike the CVD process in the presence of H_2_ where the ligands are converted to their fully reduced and thus volatile analogues [19], thermal reactions following electron exposure in the presence of H_2_O do not remove the cyclopentadienyl ligands from the deposit as such or in a reduced form. In fact, not only MeCpH but also its reduced analogues 1-methylcyclopentene and methylcyclopentane have a medium intense fragment with *m*/*z* 39 [32] and would be expected to desorb at a similar temperature as MeCpH. In conclusion, the cyclopentadienyl ligands must have been further decomposed in the present experiments.

### Water- and electron-induced degradation of a FEBID-like deposit produced from MeCpPtMe_3_

**Figure 8:**
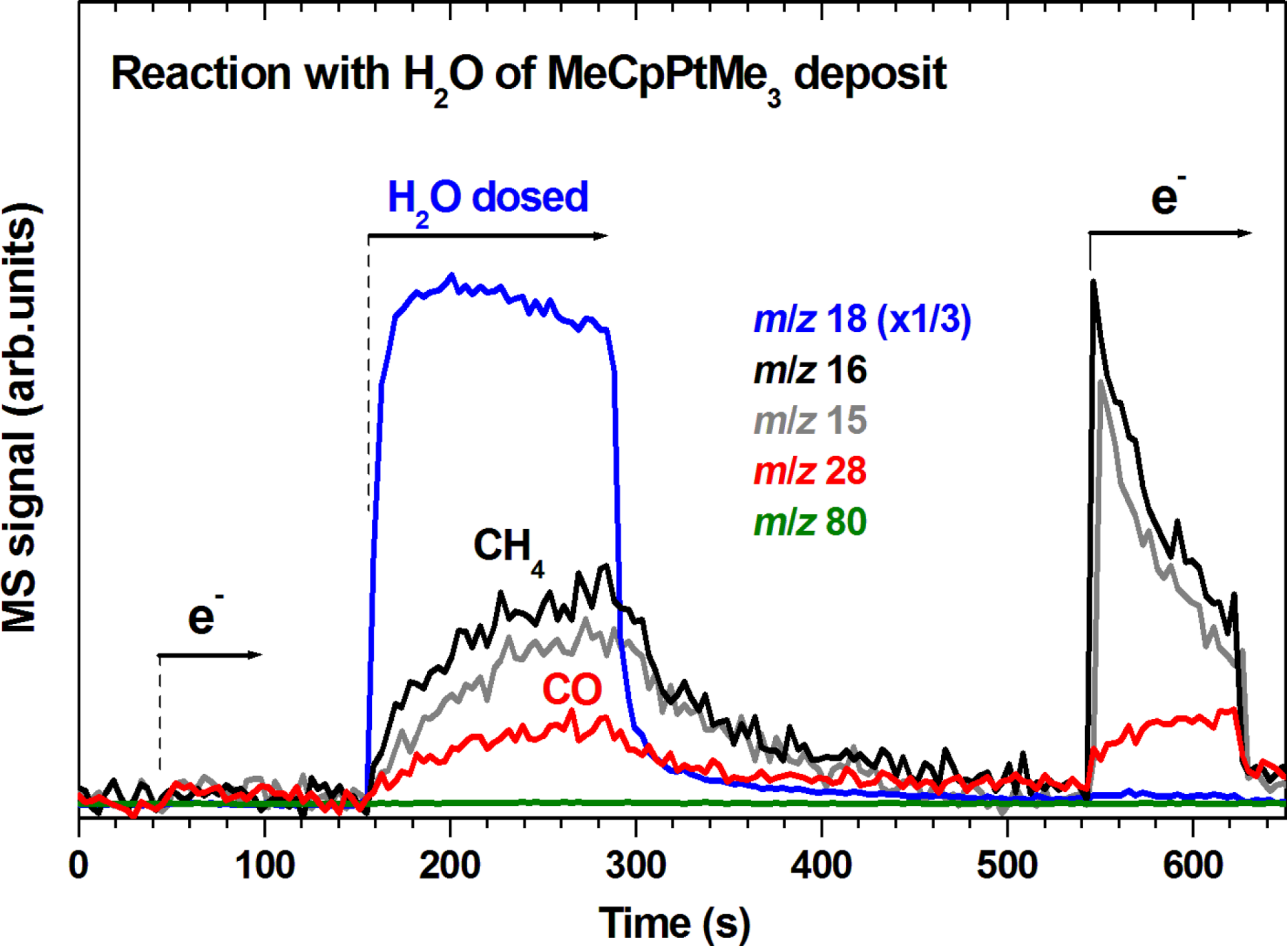
Desorption of CH_4_ (*m*/*z* 15 and 16) and CO (*m*/*z* 28) from a deposit produced from more than 500 monolayers of MeCpPtMe_3_ deposited successively on a Ta substrate held at 108 K and decomposed by electron irradiation at *E*_0_ varying between 19 eV and 31 eV followed in each deposition by annealing to 450 K to remove remaining intact precursor molecules. The *m*/*z* ratio 18 serves to monitor H_2_O leaked into the chamber or possible desorption of H_2_O from the surface during subsequent electron exposure. The thick deposit was held at 108 K and first exposed to electron irradiation at *E*_0_ = 19 eV (1 mC/cm^2^) (between 40 s and 100 s) where lack of ESD gives evidence of complete precursor degradation. H_2_O was then leaked into the chamber (between 155 s and 280 s) leading to surface reactions and consequent desorption of CH_4_ (identified from the characteristic intensity ratio for *m*/*z* 16 and *m*/*z* 15 of 1:0.9 [10]) and CO (*m*/*z* 28). Finally, the deposit was again irradiated at *E*_0_ = 19 eV (1 mC/cm^2^) between 545 s and 630 s, now leading to ESD of CH_4_ and CO. Included is also *m*/*z* 80 which is representative of MeCpH [32] thus ruling out any noticeable desorption of the MeCp ligand as a result of possible protonation.

The FEBID deposits produced from MeCpPtMe_3_ and used in previous purification experiments by post-deposition electron-beam treatment in the presence of water vapour had thickness up to 300 nm, of which the topmost 45 nm were fully purified [12]. Taking the effective diameter of 0.96 nm of an MeCpPtMe_3_ molecule [33] and considering that condensed precursor layers lose organic material and densify during degradation, a 30 monolayer condensed precursor layer would yield a deposit that is significantly thinner than such a typical FEBID deposit. To mimic a thick deposit, 30 monolayer films of MeCpPtMe_3_ were thus repeatedly deposited and exposed to electron irradiation at *E*_0_ varying between 19 eV and 31 eV without intermittent sputter cleaning. Irradiation was performed for each deposition cycle until the formation of volatile CH_4_ had ceased. Remaining intact precursor molecules were removed from the surface by thermal annealing to 450 K. The annealing step also initiates the thermal reactions that contribute to deposit formation in an actual FEBID process as obvious from our TDS data shown in Figure 4 and Figure 7. In total, more than 500 monolayers of the precursor were thus deposited on the surface so that the resulting deposit had an estimated thickness in the range of a typical FEBID nanostructure [12]. This deposit was then used for a desorption experiment to study the reactions occuring during a water-assisted purification process (Figure 8). We note that due to the necessity of cryogenic conditions imposed by our experiments, this process of deposit formation is not performed under the typical steady-state conditions of an electron-limited FEBID process. It is, however, similar in the sense that the precursor is replenished after each individual deposit formation cycle.

Prior to the start of the experimental period shown in Figure 8, the thick deposit has been subject to a few cycles of deposition of H_2_O, electron exposure, and annealing to 450 K. Figure 8 thus represents the chemistry during an ongoing deposit purification process. Following the previous treatment and shown as a first step in Figure 8, the lack of ESD from the precursor layer was verified by an electron irradiation period between 40 s and 100 s. At this stage, volatile species that were formed during previous purification cycles had been thermally desorbed prior to electron exposure and the irradiation step clearly did not produce further volatile products. Between 155 s and 280 s, vapour of H_2_O was leaked into the chamber and thus allowed to condense on or penetrate the deposit still held at 108 K. This induced reactions in the deposit as obvious from the onset of desorption of CH_4_ and CO at 155 s and increasing desorption rate afterwards. This shows that, although the previous electron exposure has not removed organic material, it has nonetheless activated the deposit. The deposited material thus thermally reacted with H_2_O even at cryogenic temperature. The resulting reactions are more obvious from the thick deposit (Figure 8) than from a thin degraded layer (Figure 5 and Figure 6) most likely because the thick layer can maintain a larger number of activated species. Desorption of products decreased again when the supply of H_2_O vapour was stopped indicating again that H_2_O is the rate limiting factor under the given conditions. After desorption had completely ceased, a second electron irradiation cycle was applied to the deposit. This led to immediate desorption of more CH_4_ but subsequent decay (Figure 8) similar to the ESD of CH_4_ observed upon degradation of a fresh precursor layer (Figure 3). Further CO was also produced but, in contrast to CH_4_, its desorption rate increased during ongoing irradiation. This indicates again that CO was not trapped in the film as such but produced during electron exposure from an intermediate species.

Figure 8 shows clearly that thick FEBID like deposits release substantial amounts of CH_4_ and CO under irradiation in the presence of water. The results indicate that, following extensive electron exposure, a FEBID deposit produced from MeCpPtMe_3_ may still be reactive and thus change its composition when it later encounters vapour of H_2_O. We note that the rate limiting effect of H_2_O was also visible during a later stage of the experiment where the vapour pressure of H_2_O above the deposit and thus the supply of H_2_O to the deposit was increased during electron exposure. This also led to an increase in product desorption rates (Supporting Information File 1, Figure S4).

It is well known that FEBID processes based on MeCpPtMe_3_ produce a nanogranular material consisting of Pt nanoparticles embedded in a carbonaceous matrix [12]. We note that the repeated cycles of applying H_2_O to the deposit followed by electron irradiation and annealing in fact has removed noticeable amounts of carbon. This was visible in AES data (Supporting Information File 1, Figure S5). While prior to purification, the signals of Pt embedded in the dominant amount of carbonaceous material were not visible, they clearly emerged after repeated purification cycles, underlining that these have in fact removed at least part of the carbon content in the present model experiments.

### Possible reaction pathways leading to formation of CO

The observed formation of CO as well as the degradation of the MeCp ligand that is obvious from the lack of MeCpH desorption in TDS experiments (Figure 7 and Figure 8) must both involve multiple bond dissociation or rearrangement. Catalytic reactions are one possibility to explain this result. A catalytic action of Pt in complexes resulting from electron-induced loss of ligands from MeCpPtMe_3_ provides a conceivable scenario for the formation of CO upon exposure of the deposit to H_2_O without electron irradiation. Pt(II) species are possibly formed when ligands are dissociated from the precursor. Such complexes are capable of inserting into C–H bonds [34], a reaction that is fundamental to the known catalytic action of Pt for the oxidation of organic compounds in aqueous solution [30]. Such reactions might also contribute to the degradation of the MeCp anionic ligand and thus prevent its neutral form MeCpH from being detected. Also, catalytic reactions may proceed on the surface of Pt nanoparticles [22–23] as produced during deposit formation [12]. This idea is supported by the previous gas-phase study that included not only PtCH_2_^+^ but also small clusters Pt_n_CH_2_^+^ and reported that small Pt_n_^+^ clusters have significantly higher activity with respect to oxidation of CH_4_ by H_2_O than Pt^+^ [23]. Within this scenario, the more obvious release of volatile products without electron irradiation from the thick deposit (Figure 8) as compared to the decomposed thin multilayer film (Figure 5 and Figure 6) can be explained as an effect of a larger number of nanoparticles. However, detailed microscopic studies would be required to support this interpretation.

Alternatively, the formation of CO during electron exposure in the presence of H_2_O can be explained by an electron-driven reaction. In fact, this type of chemistry can explain both the degradation of the MeCp ligand as well as the formation of CO. As previously shown, CO is produced during electron-driven degradation of methanol which is the smallest alcohol [35]. Alcohols, on the other hand, can be formed through electron-initiated reactions. This was demonstrated for a condensed mixture of ethylene (C_2_H_4_) and H_2_O [31]. Electron exposure initiated addition of H_2_O to the CC double bond of C_2_H_4_ yielding ethanol (C_2_H_5_OH). The reaction proceeds through both an ionization-driven reaction and a complex mechanism involving DEA [31]. This led us to the hypothesis that electron irradiation may also drive addition of H_2_O to the MeCp ligand of MeCpPtMe_3_ or to MeCpH released from the precursor upon electron-driven proton transfer (see above). The resulting alcohol would then further decompose to release CO.

Beside formation of an alcohol, other types of reactions are conceivable. Representative isomers of the different types of reaction products are listed in Table 1. In fact, electron-induced CH bond cleavage releases atomic hydrogen that, in a condensed phase, can add to CC double bonds. This can induce a sequence of reactions that leads to formation of the saturated analogue of an initially unsaturated hydrocarbon molecule [36]. In the case of MeCpH, such a reduction of one of the two double bonds would lead to the different isomers of methylcyclopentene. Also, it is known that single electron oxidation of dienes significantly reduces the activation barrier towards formation of the Diels–Alder dimer [37] so that this product is also anticipated under electron exposure at *E*_0_ above the ionization threshold. Table 1 also includes characteristic mass spectrometric signals of these different types of compounds as a basis for the analysis of our experimental product identification.

**Table 1:** Anticipated products of electron-induced reactions of pure MeCpH or of MeCpH in presence of H_2_O.

MeCpH		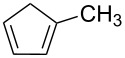	
Reaction	Electron-induced hydration [31]	Reduction by atomic hydrogen resulting from electron-induced CH bond cleavage of adjacent molecules [36]	Electron-induced dimer formation (Diels–Alder-type reaction) [37]
Anticipated reaction products	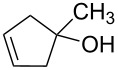	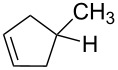	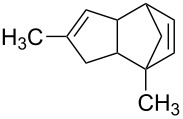
Characteristic MS fragments	*m*/*z* 83 [M−CH_3_]^•+^ [38]	*m*/*z* 67 [M−CH_3_]^•+^ [32]	*m*/*z* 81 [MeCpH_2_]^+^ [this work]

**Figure 9:**
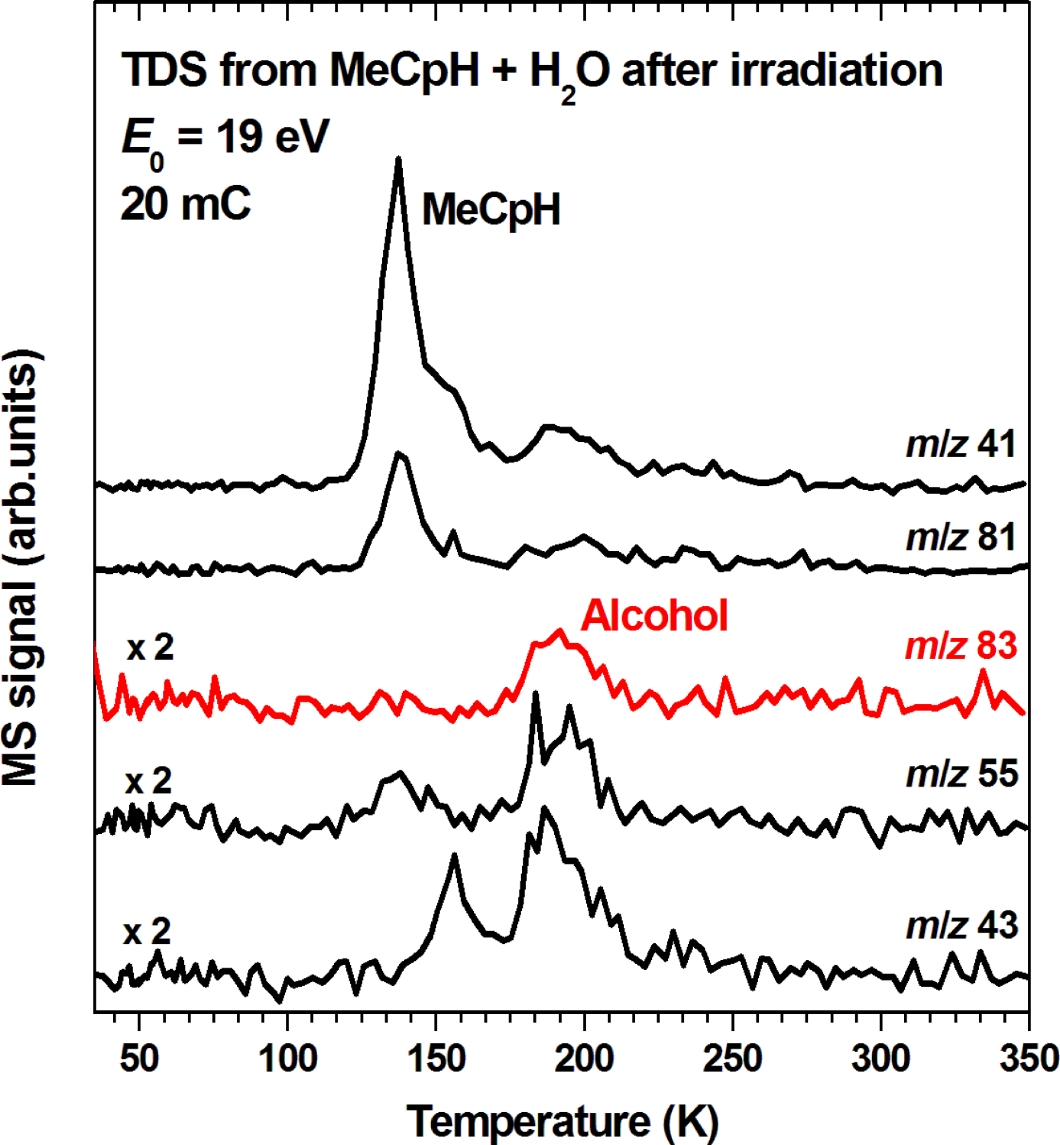
Thermal desorption spectra from a mixed condensed layer of MeCpH and H_2_O (1:1) with estimated thickness corresponding to 20 monolayers on a Au substrate recorded after an electron exposure of 4 mC/cm^2^ at *E*_0_ = 19 eV with the substrate held at 35 K. See text and Table 1 for explanations on selection of *m*/*z* ratios shown here.

To investigate if an alcohol is indeed formed, a mixed condensed layer of methylcyclopentadiene (MeCpH) and H_2_O was irradiated and subsequently analyzed by TDS. According to literature, the mass spectrum of the anticipated alcohol that would result from addition of H_2_O to MeCpH, represented by 1-methyl-2-cyclopenten-1-ol is dominated by a signal at *m*/*z* 83 [38]. Other signals near and above this mass were small while signals below *m*/*z* 79 were not reported. Figure 9 shows TDS scans recorded after electron exposure to the mixed layer of MeCpH and H_2_O including the anticipated *m*/*z* 83. In fact, a desorption signal with maximum around 190 K is seen in the TDS scan at *m*/*z* 83 as well as in scans recorded at *m*/*z* 55, and *m*/*z* 43. We note that the experiment was repeated several times to screen all conceivable mass fragments but only those that showed a desorption signal that apparently relates to the anticipated product are shown here. *m*/*z* 41 and *m*/*z* 81 are included because they are minor fragments in the EI mass spectrum of MeCpH [32] as confirmed by the characteristic desorption signal with maximum at 137 K (compare Supporting Information File 1, Figure S6).

**Figure 10:**
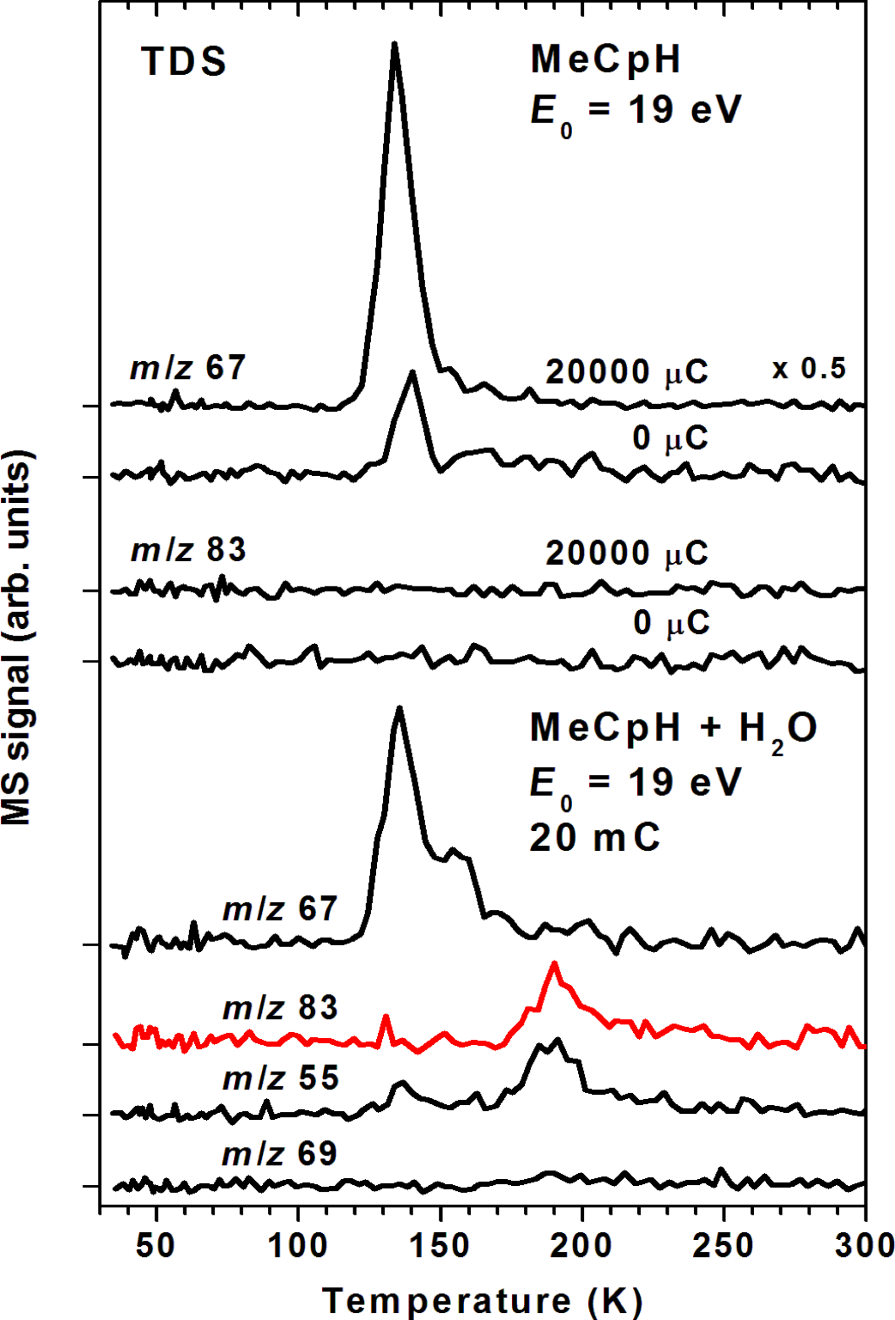
Thermal desorption spectra from condensed layers of MeCpH with estimated thickness corresponding to 15 monolayers on a Au substrate recorded before and after an electron exposure of 4 mC/cm^2^ at *E*_0_ = 19 eV with the substrate held at 35 K (top) and of a mixed condensed layer of MeCpH and H_2_O (1:1) with estimated thickness corresponding to 20 monolayers on the same substrate recorded after an electron exposure of 4 mC/cm^2^ at *E*_0_ = 19 eV with the substrate held at 35 K (bottom). See text and Table 1 for explanations on selection of *m*/*z* ratios shown here.

To support the assignment of the 190 K desorption signal to the anticipated alcohol, other conceivable products must be ruled out. The mass spectra of all isomers of methylcyclopentene are dominated by a fragment with *m*/*z* 67 [32]. In fact, an *m*/*z* 67 desorption signal emerges at 135 K upon electron exposure of a pure condensed layer of MeCpH while *m*/*z* 83 is not observed under these conditions (Figure 10, top). This clearly shows that the *m*/*z* 83 desorption signal at 190 K is not due to reduced forms of MeCpH. In contrast, both the product with one reduced double bond (*m*/*z* 67 at 135 K) and the desorption signal at 190 K in *m*/*z* 83 and as well as *m*/*z* 55 are observed when MeCpH mixed with H_2_O is irradiated (Figure 10, bottom) showing that reduction of MeCpH is a concurrent reaction to addition of H_2_O yielding an alcohol. At the same time, the absence of a noticeable desorption signal in the *m*/*z* 69 curve which is one of the most intense fragments of methyl cyclopentane [32] rules out the hydrogenation of both double bonds.

While the methylcyclopentene isomers desorb at the same temperature as MeCpH itself, the higher desorption temperature of 190 K in the *m*/*z* 83 curve points to the formation of a larger product. However, a mass spectrum of the MeCpH dimer that was recorded as reference does not show a signal at *m*/*z* 83 but a medium intensity fragment at *m*/*z* 81. Such a significantly more intense *m*/*z* 81 signal is not seen around 190 K (see Figure 9). These arguments rule out an assignment of the 190 K desorption peak to the MeCpH dimer and thus support that the anticipated alcohol is in fact produced under electron exposure of MeCpH in the presence of H_2_O.

**Figure 11:**
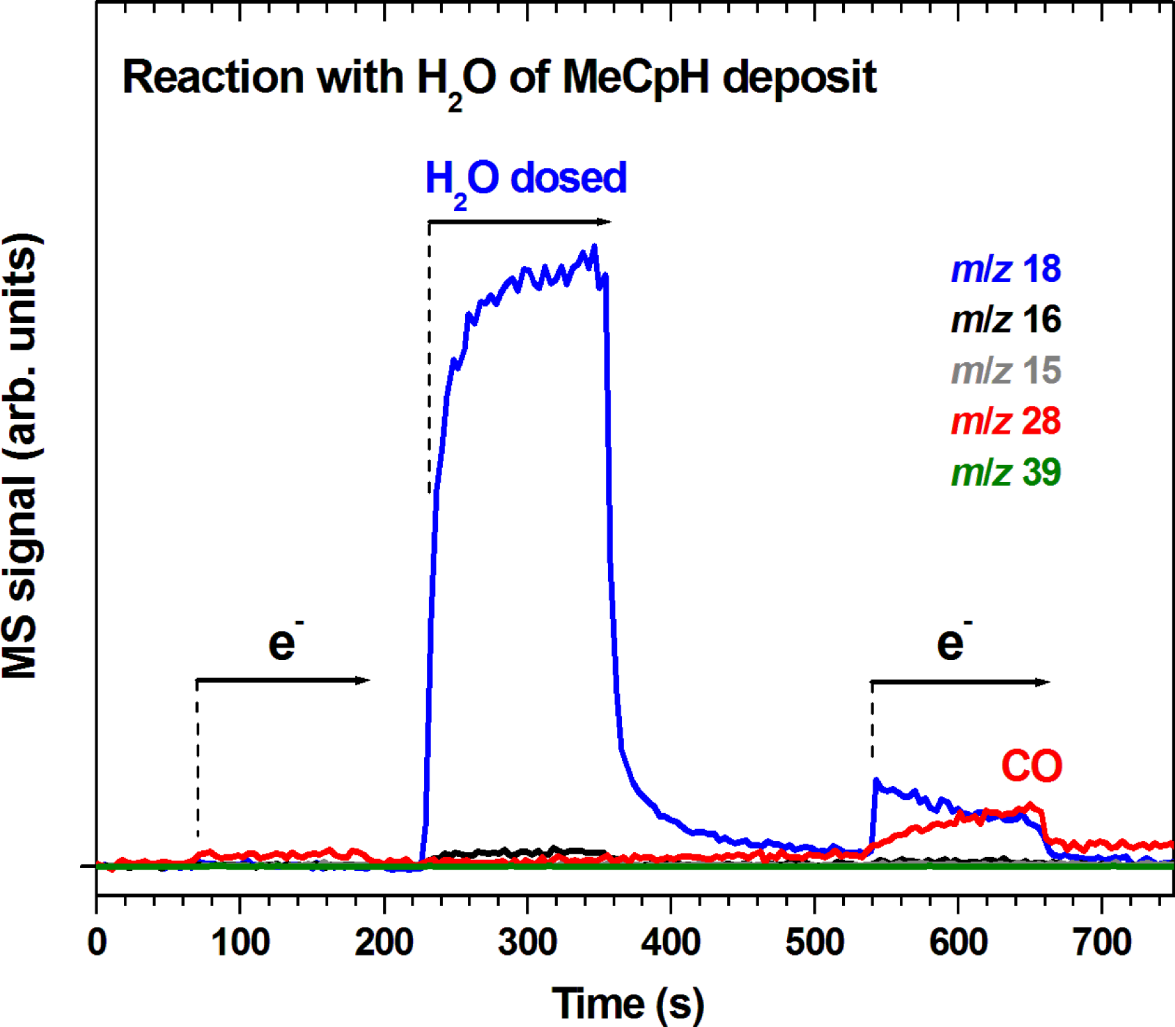
MS signals recorded during electron exposures (e^−^) and leaking of H_2_O (H_2_O) onto a deposit that has been prepared by dosing a large amount of MeCpH onto a Ta substrate held at 105 K and simultaneous electron exposure at *E*_0_ = 31 eV to build up a crosslinked carbonaceous layer. Remaining MeCpH was removed by annealing to room temperature prior to cooling the substrate down again and starting the experimental sequence shown here. During leaking of H_2_O, an amount of vapour corresponding to 2–3 monolayers was applied. During the two electron irradiation periods, electron exposures of 2 mC/cm^2^ each were applied at *I*_p_ = 17 µA/cm^2^ at *E*_0_ = 31 eV. The *m*/*z* ratios 18, 16, 15, and 28 were recorded to monitor leaking of H_2_O as well as possible formation of CH_4_ and CO. The *m*/*z* ratio 39 was included to show that desorption of MeCpH is negligible.

The results show that addition of H_2_O to unsaturated hydrocarbon species in the deposit likely leads to alcoholic species which are likely to release CO during electron exposure [35]. To show that electron-induced reactions with H_2_O in fact convert carbon to CO in a deposit produced from MeCpH, a large amount of the compound was slowly dosed onto the Ta substrate held at 105 K. During dosing, the condensing layers were continuously irradiated with electrons at *E*_0_ = 31 eV to build up a crosslinked carbonaceous layer. Remaining MeCpH was then removed by annealing to room temperature. After cooling down again, as shown in Figure 11, a first ESD experiment was performed leading to negligible production of volatile species. After irradiation was stopped, H_2_O was dosed onto the deposit yielding again no significant amounts of volatile products. However, a second period of electron exposure (between 540 s and 660 s) led not only to immediate desorption of H_2_O that had condensed on or diffused into the deposit. Also, desorption of CO was observed with increasing intensity during irradiation giving again evidence that an intermediate was formed that decomposed under electron exposure to yield CO. It is very likely that addition of H_2_O to unsaturated molecular groups in the carbonaceous material resulting in formation of alcoholic species is also involved in this case. Such reactions can thus make contributions to the removal of carbon during deposit purification processes assisted by H_2_O.

## Conclusion

The present study uses a combination of different desorption experiments to obtain a deeper insight in the chemistry underlying the recently reported water-assisted purification of FEBID deposits produced from MeCpPtMe_3_ [12]. This approach combines thermal and electron-induced desorption spectrometry with experiments that monitor volatile products being formed upon contact of deposited material with vapour of H_2_O. The experiments thus differentiate between electron-induced reactions and contributions of thermal chemistry to the formation of volatile decomposition products during precursor degradation. A comparison of MS intensities obtained during electron-stimulated desorption (ESD) and in a post-irradiation thermal desorption spectrometric (TDS) experiment allows us to estimate that more CH_4_ is released upon temperature increase than during irradiation of the precursor at liquid nitrogen temperature. These thermal processes must be taken into account when comparing the results of a surface science model study to an actual FEBID process.

Based on the present results in connection with previous experiments we can safely confirm that CH_4_ and not CH_3_ is released from the condensed layer of MeCpPtMe_3_ during electron-induced fragmentation. This finding suggests that efficient reaction channels exist in the condensed layer that either produce CH_4_ or retain CH_3_ in the deposit.

Regarding the water-assisted purification of FEBID deposits produced from MeCpPtMe_3_, the present experiments show that the combined action of H_2_O and electron exposure is required for an efficient removal of organic material at cryogenic temperature. However, evidence was obtained that the reaction rates are limited by the available supply of H_2_O under the given vacuum conditions. We propose that ionization of H_2_O and subsequent proton transfer and formation of OH radicals is an essential reaction step in deposit purification chemistry. The fact that OH radicals are involved can also rationalize the depth dependence of the efficiency of electron-driven deposit purification processes assisted by H_2_O as reported previously [12].

Desorption of CO that is produced beside CH_4_ during the water-assisted purification reactions is typically delayed as compared to CH_4_. This implies that CO is released from an intermediate product. We propose that electron-induced reactions lead to alcoholic species as intermediates. These are formed by addition of H_2_O to unsaturated molecular groups in the carbonaceous material resulting from decomposition of the precursor ligands. Further electron-induced decomposition of this alcohol then yields CO. This also accounts for the finding that the cyclopentadienyl ligand cannot be desorbed from the deposit as an intact unit. We propose that such reactions are relevant to the removal of carbon during deposit purification processes assisted by H_2_O and also to the recently reported electron-induced etching of graphene using an ice layer as resist [17]. Contributions from catalytic chemistry on Pt complexes and/or Pt nanoparticles are also conceivable. However, further studies using, in particular, quantum chemistry are needed to gain more insight in such reactions as well as in the properties of reactive intermediates involved in precursor decomposition and deposit purification processes.

Finally, the present results also suggest that under conditions relevant to the actual technical process, FEBID deposits may react with H_2_O even in the absence of electron irradiation. Specific attention should thus be paid to stability under moist conditions of devices fabricated by FEBID from MeCpPtMe_3_.

## Experimental

All experiments were performed in an ultrahigh vacuum (UHV) chamber [39] with a base pressure of about 10^−10^ mbar. In all experiments, multilayer films of MeCpPtMe_3_ were condensed on a polycrystalline Ta sheet held between 105 K and 110 K by liquid N_2_ cooling. The condition of the Ta substrate was monitored by Auger electron spectroscopy (STAIB DESA 100). Prior to an experiment, the substrate was sputter-cleaned using Ar^+^ ions at 3 keV until the Auger signals of the underlying Ta were clearly visible. Immediately before the precursor deposition, adsorbed volatile compounds from the residual gas were further removed by annealing to 450 K through resistive heating of two thin Ta ribbons spot-welded to the thicker Ta sheet.

To produce the precursor films, vapours of MeCpPtMe_3_ were introduced via a gas handling manifold consisting of precision leak valves and a small calibrated volume where the absolute pressure is measured with a capacitance manometer. For each film deposition, a calibrated amount of vapour was leaked via a stainless steel capillary opening onto the metal substrate. Experiments on methylcyclopentadiene (MeCpH) were performed on a polycrystalline Au sheet held at 35–38 K by a closed-cycle helium refrigerator (Leybold Vacuum). This substrate was cleaned between the experiments by annealing to 400 K again using two thin Ta resistive heating ribbons spot-welded to the thicker Au sheet.

The film thickness of MeCpPtMe_3_ layers was estimated by thermal desorption spectrometry (TDS) of films with increasing coverage. The data recorded at 16 amu (Supporting Information File 1, Figure S7) show a broad and weak desorption signal with maximum around 230 K which rapidly saturates and is therefore ascribed to the monolayer. A second peak with maximum at 210 K starts to increase upon saturation of the monolayer peak and is hence attributed to the successive layers no longer in contact with the substrate. The multilayer desorption temperature agrees with values observed previously on Au(110) [10]. In the same way, the monolayer coverage of MeCpH was estimated (Supporting Information File 1, Figure S6). In all experiments, multilayer films with a thickness of 30 layers (±30%) were prepared. In purification and control experiments, typical quantities of H_2_O vapour leaked into the chamber corresponded to those used for deposition of MeCpPtMe_3_ and MeCpH (see also Figure captions).

Desorption experiments were performed by use of a quadrupole mass spectrometer (QMS) residual gas analyser (Stanford, 300 amu) with electron impact ionization at 70 eV. The sample temperature was measured using a type E thermocouple press-fitted to the Ta or Au substrate. For electron-stimulated desorption (ESD) isothermal experiments, the sample was kept at the lowest attainable temperature and exposed to electron irradiation using a commercial flood gun (SPECS FG 15/40). This electron source delivers electrons with tunable kinetic energy (*E*_0_) at an estimated resolution of the order of 0.5–1 eV and currents as measured at the substrate (*I*_p_) of the order of up to 150 µA for an irradiated area of 5 cm^2^. Desorption of volatile species was also monitored, again under isothermal conditions, upon dosing of H_2_O, either without or with simultaneous electron exposure. Thermal desorption spectrometry (TDS) was performed by applying a temperature ramp of 1 K/s to the sample.

MeCpPtMe_3_ was purchased from STREM and ACROS, both at a stated purity of 99%, and degassed by repeated freeze-pump-thaw cycles. MeCpH was a mixture of isomers (1-, 2-, and 3-methylcyclopenta-1,3-diene) and prepared from the dimer by thermal cracking above 172 °C. It was kept below −20 °C in the sample reservoir throughout the experiments to prevent the reverse reaction to the dimer.

## Supporting Information

File 1 Additional experimental data.
