# Supplementary material for: Electron-driven and thermal chemistry during water-assisted purification of platinum nanomaterials generated by electron beam induced deposition
Source: Beilstein J Nanotechnol. 2018 Jan 8;9:77–90. doi: 10.3762/bjnano.9.10 (PMC5789382; doi:10.3762/bjnano.9.10)
Supplement: File 1 — Additional experimental data. [file Beilstein_J_Nanotechnol-09-77-s001.pdf]

Supporting Information

for

Electron-driven and thermal chemistry during water-assisted purification of platinum nanomaterials generated by electron beam induced deposition

Ziyan Warneke[1,‡], Markus Rohdenburg[1,‡], Jonas Warneke[1,2], Janina Kopyra[3] and Petra Swiderek[*1]

Address: [1]University of Bremen, Faculty 2 (Chemistry/Biology), Institute of Applied and Physical Chemistry, Leobener Straße / NW 2, Postfach 330440, D-28334 Bremen, Germany, [2]Pacific Northwest National Laboratory, Physical Science Division, Richland, WA, USA and [3]Siedlce University, Faculty of Sciences, 3 Maja 54, 08-110 Siedlce, Poland

Email: Petra Swiderek - swiderek@uni-bremen.de

* Corresponding author

‡ Both authors contributed equally to this publication

Additional Experimental Data

Thermal desorption spectrum of $MeCpPtMe_3$ on Ta

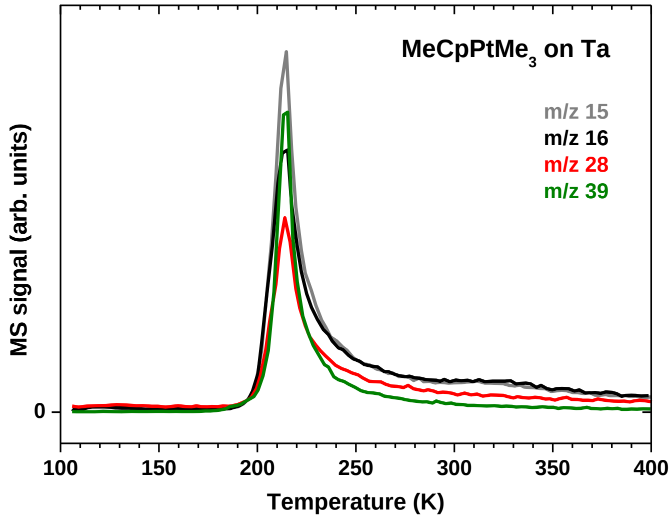

Figure S1: Thermal desorption spectra recorded from a 30 layer film of $MeCpPtMe_3$ deposited on a Ta sheet held at 105 K. The *m*/*z* ratios 15 (CH_3^+), 16 (($CH_4^{\bullet+}$), 28 ($C_2H_4^{\bullet+}$), and 39 ($C_3H_3^+$) represent some of the positive ion fragments of the precursor that are produced by electron impact ionization in the ionizing unit of the quadrupole mass spectrometer.

ESD during initial stages of irradiation of a multilayer film of $MeCpPtMe_3$ on Ta

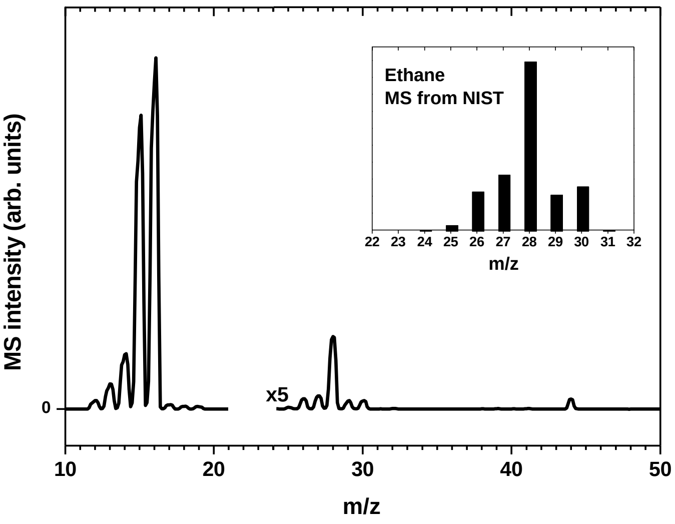

Figure S2: Mass spectrum recorded during the initial stage of electron irradiation of a 30 layer film of $MeCpPtMe_3$ deposited on a Ta sheet held at 105 K such as shown in Figure 3. The *m/z* ratios 12 ($C^{\bullet+}$), 13 (CH^+), 14, ($CH_2^{\bullet+}$), 15 (CH_3^+), and 16 (($CH_4^{\bullet+}$) are assigned to desorption of CH_4. The group of signal ranging from *m/z* 26 to *m/z* 30 gives evidence of desorption of minor quantities of ethane (C_2H_6) as seen by comparing with a literature spectrum [NIST Mass Spec Data Center, S.E. Stein, director, "Mass Spectra" in NIST Chemistry WebBook, NIST Standard Reference Database Number 69, Eds. P.J. Linstrom and W.G. Mallard, National Institute of Standards and Technology, Gaithersburg MD, 20899, doi:10.18434/T4D303, (retrieved June 13, 2017)] included in the inset. Excess intensity as compared to the spectrum of C_2H_6 at m/z 28 amounting to roughly half of the total signal height is ascribed to CO due to the close agreement of the other signals with the literature spectrum of C_2H_6.

ESD during irradiation of a multilayer film of MeCpPtMe$_3$ on Ta in contact with H_2O

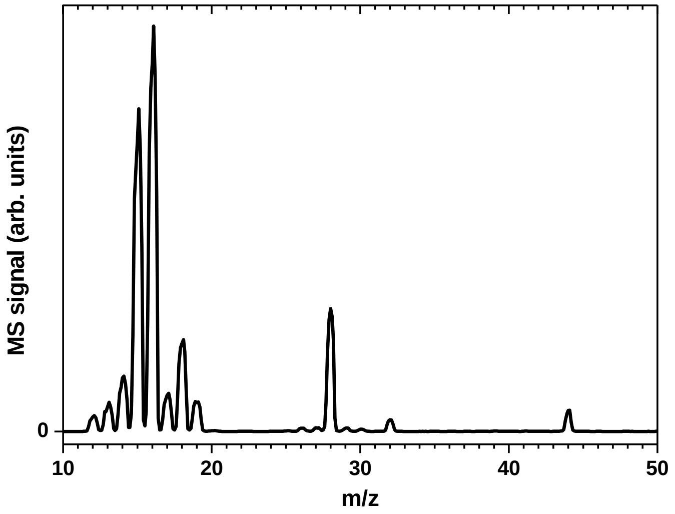

Figure S3: Mass spectrum recorded during electron irradiation at E_0 = 31 eV of a previously electron-degraded 30 layer film of MeCpPtMe$_3$ deposited on a Ta sheet held at 105 K onto which H_2O has been dosed (see Figure 5). The *m*/*z* ratios 12 ($C^{\bullet +}$), 13 (CH^+), 14, ($CH_2^{\bullet +}$), 15 (CH_3^+), and 16 (($CH_4^{\bullet +}$) are assigned to desorption of CH_4 while *m*/*z* 18 reveals desorption of H_2O. Compared to Figure S2, the presence of H_2O leads to a strong increase of the signal at *m*/*z* 28 providing evidence that CO is formed.

Water- and electron-induced degradation

# of a FEBID-like deposit produced from $MeCpPtMe_3$

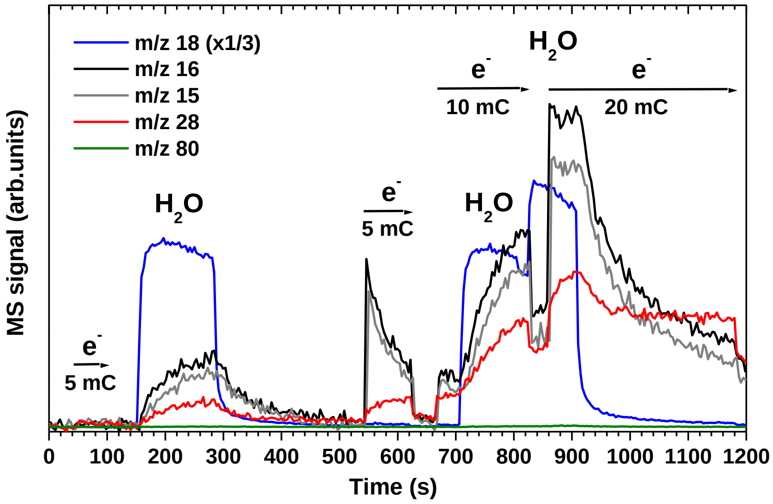

Figure S4: Extended plot of the data shown in Figure 7. Desorption of CH_4 (*m*/*z* 15 and 16) and CO (*m*/*z* 28) from a deposit produced from more than 500 monolayers of $MeCpPtMe_3$ deposited successively on a Ta substrate held at 108 K and decomposed by electron irradiation at E_0 varying between 19 eV and 31 eV followed in each deposition by annealing to 450 K to remove remaining intact precursor molecules. *m*/*z* 18 serves to monitors H_2O leaked into the chamber or possible desorption of H_2O from the surface during subsequent electron exposure. The thick deposit was held at 105 K and first exposed to electron irradiation at E_0 = 19 eV (5 mC) (between 40 s and 100 s) where lack of ESD gives evidence of complete precursor degradation. H_2O was then leaked into the chamber (between 155 s and 280 s) leading to surface reactions and consequent desorption of CH_4 and CO. The deposit was again irradiated at E_0 = 19 eV (5 mC) between 545 s and 630 s, now leading to ESD of CH_4 and CO. H_2O was leaked again starting at 700 s with leaking rate and thus chamber pressure increased at 820 s. During simultaneous electron exposures (10 mC between 670 s and 820 s, 20 mC between 860 s and 1180 s), the limiting effect of the supply of H_2O is clearly visible from a higher product desorption rate at higher leaking rate of H_2O. Included is also *m*/*z* 80 which is representative of both $MeCpPtMe_3$ and MeCp thus ruling out any noticeable desorption of the precursor or of its MeCp ligand.

Auger Electron Spectroscopy of Pt

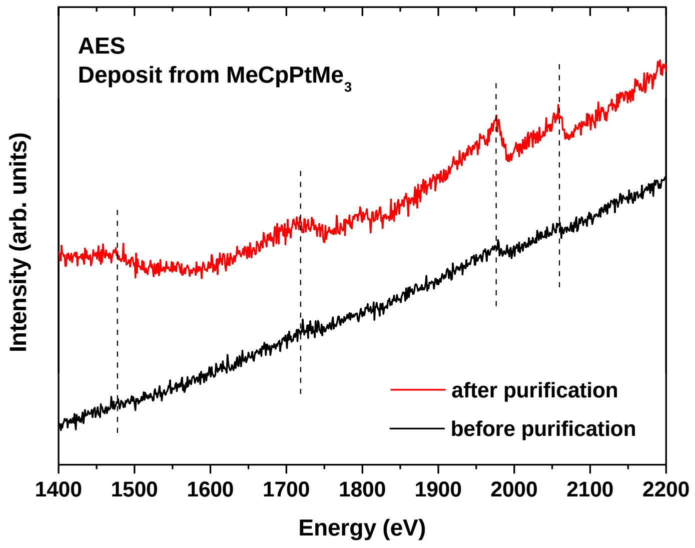

Figure S5: Auger electron spectra in the range of the Pt signals acquired at 5 keV from a deposit as shown in Figure S1 before and after several cycles of water-assisted purification. As a result of removal of organic material, signals emerge that agree closely with reference data for Pt (Energy/Sensitivity factor: 1484 eV/0.0245, 1725 eV/0.0458, 1969 eV/0.1569, 2048 eV/0.1296).

Thickness calibration for MeCpH

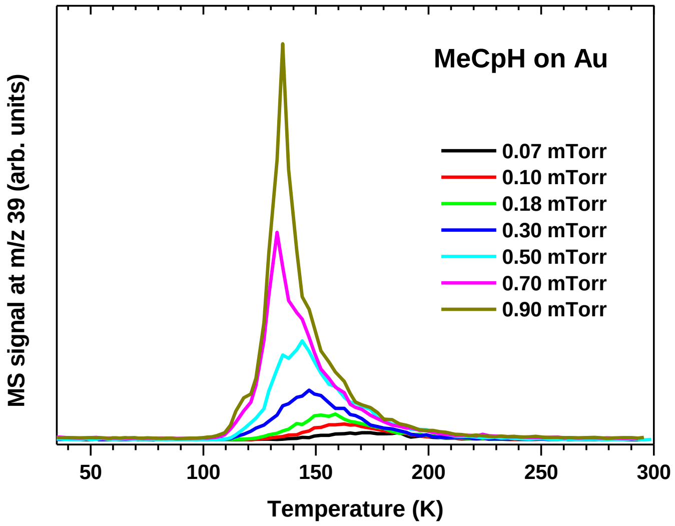

Figure S6: Representative thermal desorption spectra recorded at 39 amu for films of MeCpH deposited from increasing amounts of vapour on a Au sheet held at 35 K. The stated pressure denotes the pressure drop in the gas inlet system during leaking of the vapour into the main chamber. These data provide the estimate that an amount of vapour corresponding to a pressure drop between 0.07 mTorr and 0.10 mTorr was required for the formation of a monolayer of MeCpH.

Thickness calibration for $MeCpPtMe_3$

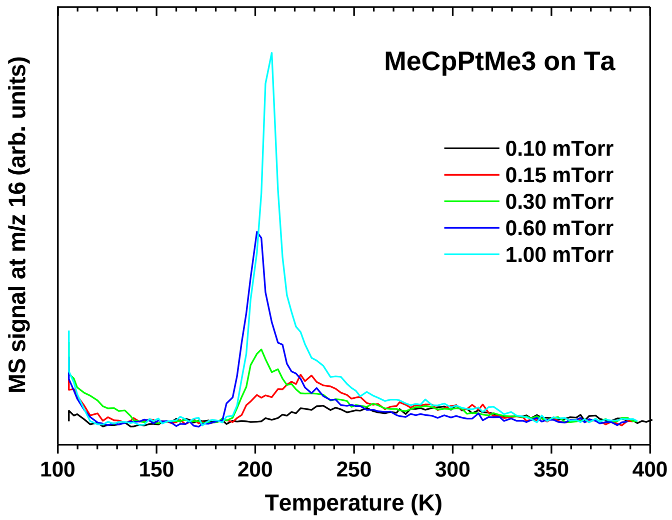

Figure S7: Representative thermal desorption spectra recorded at 16 amu for films of $MeCpPtMe_3$ deposited from increasing amounts of vapour on a Ta sheet held at 105 K. The stated pressure denotes the pressure drop in the gas inlet system during leaking of the vapour into the main chamber. These data give evidence that an amount of vapour corresponding to a pressure drop between 0.1 mTorr and 0.15 mTorr was required for the formation of a monolayer of $MeCpPtMe_3$.
